# A comprehensive study of psychological well-being and traditional Chinese medicine constitutions among model workers in Beijing

**DOI:** 10.3389/fpsyt.2024.1425757

**Published:** 2024-09-11

**Authors:** Hongli Cao, Xianyang Chen, Yige Song, Shawn Xiang Li, Hui Ma, Guosheng Zhang, Tianyu Gong, Hong Yu, Zijin Liu

**Affiliations:** ^1^ Department of Emergency Medicine, Beijing Rehabilitation Hospital, Capital Medical University, Beijing, China; ^2^ Department of medicine development, Bao Feng Key Laboratory of Genetics and Metabolism, Beijing, China; ^3^ Emergency Department, Beijing Rehabilitation Hospital, Capital Medical University, Beijing, China; ^4^ Model Worker Health Management Center, Capital Medical University, Beijing, China; ^5^ Department of Orthopedic Rehabilitation, Beijing Rehabilitation Hospital, Capital Medical University, Beijing, China

**Keywords:** traditional Chinese medicine (TCM), model workers, mental health, TCM body constitution, partial least squares path modeling

## Abstract

**Background:**

Body constitution is the foundation of Traditional Chinese Medicine (TCM), and model workers consist of a special group of workers within China. This study aims to research the relationship between the physical body constitutions based on TCM and the mental health of model workers.

**Methods:**

We recruited 314 model workers from Beijing Rehabilitation Hospital to conduct the questionnaires such as SCL-90 and CCMQ to investigate if there is an association between mental health status and TCM body constitutions. We performed a Partial Least Squares Path Modeling (PLS path modeling).

**Results:**

Our path model results revealed associations between different TCM constitution types and SCL scores, which serve as indicators of psychological well-being. Our research findings demonstrate a strong correlation between the Balanced constitution and elevated levels of psychological well-being, with a path coefficient of -0.503. In contrast, the other eight constitutional types exhibit path coefficients exceeding 0.3, indicating a tendency toward lower levels of psychological well-being. We also investigated the intricate connections between various TCM constitutional types and both mild and severe psychological well-being.

**Conclusion:**

In conclusion, the Balanced constitution continues to be closely associated with higher levels of psychological well-being, while the remaining eight body constitution types are consistently linked to lower levels of psychological well-being.

## Introduction

The relationship between an individual’s constitution, or bodily type, and disease is a crucial notion within the theory of Traditional Chinese Medicine(TCM) (1). In TCM, body constitution forms the basis of understanding health and disease, focusing on what makes a person vulnerable to specific diseases, including psychological disorders (1–4). Based on the “Classification and Determination of Constitution in TCM” published by the China Association of Chinese Medicine (CACM), which is regarded as the professional standard, people can be categorized into nine types: Balanced constitution, Qi-Deficiency constitution, Yang-Deficiency constitution, Yin-deficiency constitution, Phlegm-dampness constitution, Damp-heat constitution, Blood-stasis constitution, Qi-stagnation constitution, and Allergic constitution (4). The classification of each TCM body constitution is based on each person’s physical features, psychological or mental characteristics, physiological characteristics and more (4, 5). A Balanced constitution represents a harmonious state, achieving a balance between Yin and Yang (6, 7). People with Qi-stagnation constitution often handle stressful situations poorly and tend to be thin, with mood fluctuations, suspicion, excessive thinking, and excessive worrying (1, 6, 8). People with Blood-stasis constitution typically have dull skin, dark lips, bruise easily, are forgetful, and dislike cold environments and weather (1, 6, 8). Furthermore, people with Qi-Deficiency constitution are prone to exhaustion due to weak immunity (1, 6, 8). People with Yin-deficiency constitution have warm palms and soles, are impatient, and are extroverted (1). In contrast, people with Yang-Deficiency constitution are usually introverted, quiet, shy, and tend to have cold limbs (8). The main features of Phlegm-dampness constitution include excessive phlegm, overweight, chest tightness, and a gentle and patient nature (1, 6, 8). People with Damp-heat constitution often have oily skin, are prone to acne, have a bitter taste in the mouth, and experience difficult and sticky bowel movements (1, 6, 8). Lastly, those with Allergic constitution are inherently sensitive to certain allergens, such as pollen, odors, food, and medicines (1, 6, 8). The Constitution in Chinese Medicine Questionnaire (CCMA) consists of 60 items designed to classify individuals into one of the nine constitutions listed (9, 10).

As mentioned above, the TCM body constitutions are closely associated with not only an individual’s physical characteristics, but even their mental health characteristics. Consequently, the CCMA questionnaire may have intrinsic correlations with psychological tools like the SCL-90 (Symptom Checklist 90), which widely used in psychological assessments. The SCL-90, developed by American psychologist Derogatis et al. in 1973 is a widely recognized tool for assessing the presence and severity of mental disorders in parents (11–13). It was translated into Chinese by Wang in the 1980s and has been extensively utilized in mental health evaluations across China (12).

Model workers represent a unique demographic in China, distinguished as the most exemplary individuals within their respective professions. This group encompasses a wide range of careers, making them a diverse representation of the working population. They are known for their dedication and significant contributions to society (14). Model workers are also individual persons, and each one may possess a distinct TCM body constitution and psychological status, which can be measured and studied to gather any intrinsic associations. We recruited a total of 314 model workers from Beijing Rehabilitation Hospital, a teaching hospital of the Capital Medical University in Beijing, China between 2018 and 2019, and we conducted the questionnaires as mentioned previously, such as the SCL-90 and CCMQ. To our knowledge, similar studies focusing on this specific population have not been conducted, making our research one of the first in this area. This study aims to investigate the relationship between TCM-based physical body constitutions and the mental health of model workers, ultimately providing insights that could enhance their quality of life, health support, and overall well-being.

## Methods

### Patient recruitment

We recruited a total of 314 model workers from Beijing Rehabilitation Hospital, a teaching hospital of the Capital Medical University in Beijing, China. This study was funded by Beijing Rehabilitation Hospital and aimed to conduct a psychological health assessment of model workers in Beijing. The necessary information has been provided to the model workers by Beijing Rehabilitation Hospital, and those who meet the inclusion/exclusion criteria have been invited to participate in the study. Only model workers who have provided informed consent and meet the eligibility criteria will be included. All participants completed the questionnaire survey between 2018 and May 8, 2019. The study has obtained approval from the Ethics Committee of Beijing Rehabilitation Hospital. The inclusion criteria for this study are: (1) being a model worker, model workers refer to those who perform exceptionally well in their respective occupations. They come from various professional fields and have made significant contributions to society (14). (2) aged between 30 and 70 years. The exclusion criteria are: (1) having a mental disorder or behavioral impairment, (2) having a severe condition that prevents accurate expression of thoughts, and (3) lacking informed consent.

The study was reviewed and approved by the Ethics Committee of Beijing Rehabilitation Hospital (reference number 2018bkkyLW009), and all eligible participants signed an informed consent form.

### Data measurement

#### SCL-90

The SCL-90, developed by American psychologist Derogatis et al. in 1973 (11, 12)^8-10^, is a widely used tool for assessing the presence and severity of mental disorders in parents. It was later translated into Chinese by Wang in the 1980s.^9-10^ The scale consists of 90 items and encompasses 10 subscales, including somatization, obsessive-compulsive symptoms, interpersonal sensitivity, depression, anxiety, hostility, phobic anxiety, paranoid ideation, psychoticism, and additional items. The total average score of the SCL-90, also known as the total symptom index, reflects the overall severity of symptoms. A higher average score indicates more pronounced symptoms. The calculation method involves dividing the total score by 90. In this study, the Chinese version of the SCL-90, which has been widely cited in China for over 30 years and demonstrates good reliability and validity, was utilized. The total scale exhibits a high Cronbach’s α coefficient of 0.97, while each subscale has a Cronbach’s α coefficient greater than 0.69. Furthermore, the test-retest reliability exceeds the 0.7 value^11^.

#### CCMQ

The “Constitution in Chinese Medicine Questionnaire” scale is used for the identification and determination of TCM constitutions (10), and consists of 60 items used to classify individuals into one or more of the nine body constitution (BC) types: Balanced constitution, Qi-Deficiency constitution, Yang-Deficiency constitution, Yin-deficiency constitution, Phlegm-dampness constitution, Damp-heat constitution, Blood-stasis constitution, Qi-stagnation constitution, and Allergic constitution. Each subscale item includes 7 or 8 questions, and each question is scored on a 5-point scale to calculate the raw score and the transformed score. The constitution type is determined according to the standard. If the determination result is multiple constitutions or tendencies toward multiple constitutions, and there are 2 (or more) identical highest scores, a professional TCM doctor will determine the main constitution type.

### Data analysis

Initially, a normality test is conducted on the data. If the data conform to a normal distribution, the mean and standard deviation are employed to depict the central tendency and dispersion of the data, and the Student’s t-test is utilized for intergroup comparisons. In the case where the data do not follow a normal distribution, the median is used, and the Mann-Whitney U test is applied for intergroup comparisons. For categorical variables, they are represented by the number of individuals and the percentage, and the Chi-square test is employed. Comparison between two groups was assessed by Students t-test or Mann-Whitney rank sum test (for continuous variables) and Chi-square test or Fisher’s exact test (for categorical variables). Comparison among multiple groups was assessed by one-way Analysis of Variance (ANOVA). Generalized quantified relationships of risk factors were evaluated using generalized additive models.

The correlation analysis is performed using the Spearman correlation coefficient. Partial Least Squares Path Model (PLS-PM) is a tool used for Partial Least Squares Path Modeling analysis. This method, a correlation-based Structural Equation Modeling (SEM) algorithm, expresses causal relationships through linear conditional expectations. It aims to identify the best linear predictive relationships and allows the use of latent variables to estimate complex causal relationships or predictive models.

All statistical analyses were performed using R version 3.6.3,and a *P*-value < 0.05 was considered statistically significant.

## Results

### Baseline characteristics

The total sample size was 314 individuals, with 134 females (35.1%) and 180 males (64.9%). The distribution of different traditional Chinese medicine (TCM) constitutions is as follows: Balanced constitution: 134 individuals, Yang-Deficiency Constitution: 50 individuals, Phlegm-Dampness Constitution: 31 individuals, Damp-Heat Constitution: 30 individuals, Qi-Deficiency Constitution: 22 individuals, Qi-Stagnation Constitution: 18 individuals, Allergic Constitution: 12 individuals, Yin-Deficiency Constitution: 9 individuals, and Blood-Stasis Constitution: 9 individuals.

There were no significant differences in the average ages among individuals with different TCM constitutions (*P*>0.05). However, individuals with different TCM constitutions exhibited significant differences in terms of weight and height. Those with a Yang-Deficiency Constitution had a lower average weight and height, whereas individuals with a Blood-stasis Constitution had a higher average weight and height (*P*<0.05). Similarly, concerning Body Mass Index (BMI), the Yang-Deficiency and Damp-Heat Constitutions were associated with lower values, while the Blood-Stasis Constitution was associated with a higher BMI (*P*<0.05). There were no noticeable differences in systolic and diastolic blood pressure among individuals with different TCM constitutions (**Table 1**).

**Table 1:** Demographics of individuals with different TCM constitutions.

Name	Levels	Balanced constitution (N=134)	Yang- Deficiency Constitution (N=50)	Phlegm-Dampness Constitution (N=31)	Damp-Heat Constitution (N=30)	Qi- Deficiency Constitution (N=22)	Qi- Stagnation Constitution (N=18)	Allergic Constitution (N=12)	Yin- Deficiency Constitution (N=9)	Blood-Stasis Constitution (N=9)	S-W test	*p*
Sex	Female	47 (35.1%)	25 (50%)	12 (38.7%)	5 (16.7%)	10 (45.5%)	12 (66.7%)	4 (33.3%)	5 (55.6%)	7 (77.8%)		0.004
	Male	87 (64.9%)	25 (50%)	19 (61.3%)	25 (83.3%)	12 (54.5%)	6 (33.3%)	8 (66.7%)	4 (44.4%)	2 (22.2%)		
Age	Mean ± SD	55.6 ± 8.8	54.5 ± 8.6	50.9 ± 10.5	51.6 ± 11.6	52.3 ± 10.8	51.9 ± 12.7	55.7 ± 12.0	56.0 ± 7.3	50.1 ± 6.4	0.074	0.138
Weight	Mean ± SD	71.4 ± 11.9	66.0 ± 10.2	73.4 ± 12.5	75.1 ± 11.1	66.0 ± 10.6	66.0 ± 8.2	66.6 ± 12.2	71.1 ± 11.3	76.0 ± 13.0	0.073	0.002
Height	Mean ± SD	167.4 ± 7.4	165.4 ± 8.6	165.8 ± 7.8	170.4 ± 6.5	164.0 ± 8.0	162.8 ± 6.8	167.1 ± 8.4	166.3 ± 7.8	164.9 ± 6.4	0.064	0.021
BMI	Median ± SD	25.1 ± 3.2	23.9 ± 2.5	26 ± 3.7	26.5 ± 3	24.2 ± 3.5	24.6 ± 2	24.8 ± 3.9	25.3 ± 2.5	28.1 ± 4.4	0.028	0.01
SBP	Mean ± SD	129.1 ± 14.5	126.0 ± 13.7	127.9 ± 16.9	125.2 ± 12.2	119.5 ± 16.7	127.5 ± 16.9	122.2 ± 18.5	129.8 ± 21.5	119.1 ± 16.8	0.481	0.136
DBP	Mean ± SD	78.3 ± 10.5	75.9 ± 7.6	74.6 ± 11.3	76.2 ± 7.9	72.4 ± 12.1	77.5 ± 12.1	74.4 ± 12.4	76.9 ± 10.2	73.1 ± 15.2	0.524	0.237

Data are expressed as mean(median)± SD or N (%).

### Association between TCM constitutions and mental health

Substantial differences were evident among the dimensions of the SCL-90 (Symptom Checklist-90) scale, particularly in the somatization dimension. Individuals with a Qi-Stagnation Constitution had a higher average score, while those with Balanced constitution had lower scores (*P*<0.05). Furthermore, our study reveals that only 21.64% of individuals with a Balanced constitution are diagnosed with mental health issues, while 94.44% of those with a Qi-Stagnation Constitution are diagnosed with mental health problems (**Table 2**).

**Table 2:** Assessment information for different TCM constitutions.

Name	Balanced constitution (N=134)	Yang- Deficiency Constitution (N=50)	Phlegm- Dampness Constitution (N=31)	Damp-Heat Constitution (N=30)	Qi- Deficiency Constitution (N=22)	Qi- Stagnation Constitution (N=18)	Allergic Constitution (N=12)	Yin- Deficiency Constitution (N=9)	Blood-Stasis Constitution (N=9)	S-W test	*p*
SCL-Somatization	1.3 ± 0.2	1.8 ± 0.6	1.7 ± 0.5	1.6 ± 0.4	1.8 ± 0.6	1.9 ± 0.7	1.7 ± 0.5	1.6 ± 0.4	1.9 ± 0.5	<.005	<.001
SCL-Obsessive-Compulsive Symptoms	1.4 ± 0.3	1.9 ± 0.7	1.8 ± 0.5	1.8 ± 0.5	2.0 ± 0.5	2.2 ± 0.6	2.0 ± 0.6	1.7 ± 0.2	2.0 ± 0.5	<.005	<.001
SCL-Interpersonal Sensitivity	1.3 ± 0.4	1.7 ± 0.6	1.7 ± 0.6	1.7 ± 0.4	2.0 ± 0.7	2.1 ± 0.7	1.6 ± 0.5	1.8 ± 0.4	1.8 ± 0.5	<.005	<.001
SCL-Depression	1.2 ± 0.2	1.6 ± 0.6	1.6 ± 0.5	1.5 ± 0.4	1.8 ± 0.7	2.2 ± 0.7	1.5 ± 0.4	1.6 ± 0.3	1.6 ± 0.5	<.005	<.001
SCL-Anxiety	1.2 ± 0.2	1.6 ± 0.6	1.5 ± 0.5	1.5 ± 0.3	1.7 ± 0.6	2.1 ± 0.8	1.6 ± 0.5	1.5 ± 0.2	1.6 ± 0.4	<.005	<.001
SCL-Hostility	1.2 ± 0.3	1.6 ± 0.7	1.7 ± 0.6	1.6 ± 0.4	1.7 ± 0.5	2.2 ± 0.8	1.6 ± 0.5	1.5 ± 0.3	1.6 ± 0.5	<.005	<.001
SCL-Phobic Anxiety	1.1 ± 0.2	1.3 ± 0.5	1.3 ± 0.4	1.2 ± 0.3	1.4 ± 0.6	1.5 ± 0.6	1.3 ± 0.4	1.2 ± 0.3	1.5 ± 0.4	<.005	<.001
SCL-Paranoid Ideation	1.1 ± 0.2	1.4 ± 0.6	1.5 ± 0.5	1.3 ± 0.3	1.6 ± 0.7	1.9 ± 0.8	1.4 ± 0.6	1.4 ± 0.4	1.5 ± 0.4	<.005	<.001
SCL-Psychoticism	1.2 ± 0.2	1.5 ± 0.5	1.4 ± 0.4	1.4 ± 0.4	1.6 ± 0.5	1.7 ± 0.7	1.4 ± 0.6	1.4 ± 0.3	1.5 ± 0.5	<.005	<.001
SCL-Sleep and Eating Disorders	1.4 ± 0.4	2.0 ± 0.8	1.8 ± 0.6	1.7 ± 0.5	1.8 ± 0.6	1.9 ± 0.5	1.9 ± 0.6	1.9 ± 0.5	2.0 ± 0.9	<.005	<.001
SCL-Totals	1.2 ± 0.2	1.6 ± 0.6	1.6 ± 0.4	1.5 ± 0.3	1.8 ± 0.5	2.0 ± 0.6	1.6 ± 0.4	1.6 ± 0.2	1.7 ± 0.4	<.005	<.001
Mental Health/Mental Illness	22%	56%	58%	57%	68%	94%	67%	56%	67%		

Data are expressed as median ± SD.

We conducted further analysis to investigate the correlation between different constitutions and two assessment scales. Our findings revealed that Yang-Deficiency and Qi-Deficiency constitutions exhibited a significant positive association with the SCL-90, particularly in the domains of depression, anxiety, and paranoid ideation (*P*<0.05). Conversely, the Balanced constitution demonstrated a notable negative correlation with all SCL-90 domains (*P*<0.05). In contrast, other constitutional types displayed relatively weaker or non-significant correlations with the SCL-90 (**Table 3**).

**Table 3:** Correlations between different constitutions and SCL scales.

	Somatization	Obsessive- Compulsive Symptoms	Interpersonal Sensitivity	Depression	Anxiety	Hostility	Phobic Anxiety	Paranoid Ideation	Psychoticism	Sleep and Eating Disorders
Balanced constitution	-0.283^***^	-0.363^***^	-0.219^*^	-0.353^***^	-0.248^**^	-0.117	-0.142	-0.272^**^	-0.266^**^	-0.358^***^
Yang-Deficiency Constitution	0.558^***^	0.717^***^	0.628^***^	0.697^***^	0.733^***^	0.600^***^	0.589^***^	0.572^***^	0.622^***^	0.481^***^
Phlegm-Dampness Constitution	0.628^***^	0.372^*^	0.364^*^	0.388^*^	0.283	0.450^*^	0.287	0.447^*^	0.321	0.335
Damp-Heat Constitution	0.262	0.252	0.05	0.192	0.007	-0.104	-0.069	0.334	0.178	0.337
Qi-Deficiency Constitution	0.823^***^	0.774^***^	0.882^***^	0.827^***^	0.804^***^	0.846^***^	0.853 ^***^	0.734^***^	0.783^***^	0.661^***^
Qi-Stagnation Constitution	0.750^***^	0.528^*^	0.573^*^	0.668^**^	0.664^**^	0.604^**^	0.466	0.572^*^	0.308	0.071
Allergic Constitution	0.635^*^	0.476	0.295	0.205	0.474	0.499	0.44	0.038	0.111	0.218
Yin-Deficiency Constitution	0.555	-0.236	-0.625	-0.003	-0.238	0.087	0.427	-0.624	-0.35	-0.144
Blood-Stasis Constitution	0.053	0.287	0	0.116	0.309	0.133	-0.289	0.16	0.158	0.127

* represent *p* <0.05; ** represent *p* <0.01; *** represent *p* <0.001.

**Figure 1:**
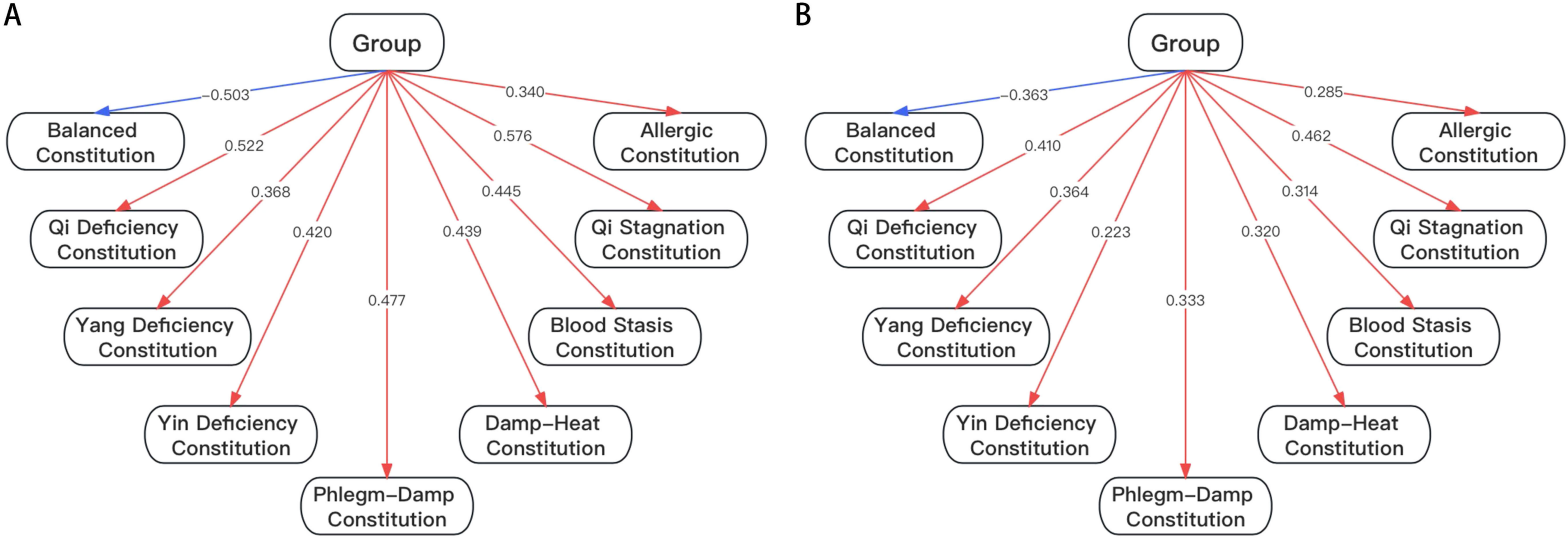
**(A)** Associations Between Different TCM Constitutional Types and Psychological Health and Illness. **(B)** Associations Between Different TCM Constitutional Types and Mild and Severe Psychological Illness.

In order to further investigate the relationships between various TCM constitutions and SCL scores, we performed a Partial Least Squares Path Modeling (PLS path modeling). Our path model results have revealed the associations between different TCM constitution types and SCL scores, which serve as indicators of psychological well-being. Our research findings demonstrate a strong correlation between the Balanced constitution and elevated levels of psychological well-being, with a path coefficient of -0.503. In contrast, the other eight constitutional types exhibit path coefficients exceeding 0.3, indicating a tendency toward lower levels of psychological well-being (*P*<0.05). We also investigated the intricate connections between the various TCM constitutional types and associations with both mild and severe psychological well-being. The results of this investigation underscore that the Balanced constitution continues to be closely associated with higher levels of psychological well-being, while the remaining eight constitutional types are consistently linked to lower levels of psychological well-being (**Figure 1**).

### In - depth analysis of SCL symptoms

**Figure 2:**
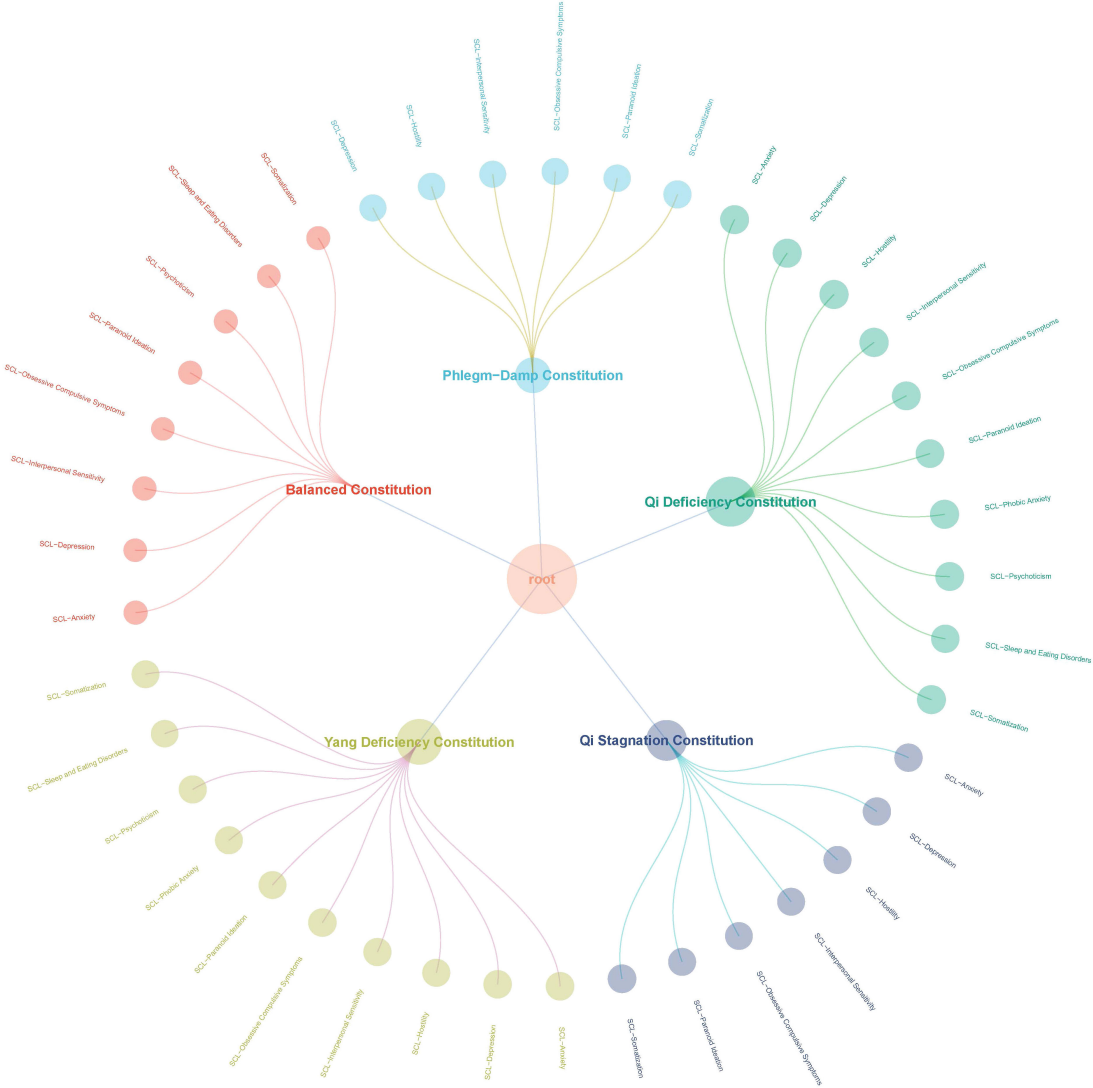
Correlation of TCM body constitution and SCL.

According to **Figure 2**, after in-depth analysis of each symptom of SCL that is significantly related, the study shows that the quality of peace is negatively correlated with all SCL. The positive correlation between Qi-Deficiency constitution and SCL was the most significant, followed by Yang-Deficiency constitution and Qi-stagnation constitution, and finally Phlegm-dampness constitution. It should be emphasized that the research results highlight the negative trend of peace quality in the relationship with various SCLs. At the same time, the positive correlation between Qi-Deficiency constitution and SCL occupies a significant position in the study, highlighting that Qi-Deficiency constitution may play an important role in the development of related symptoms.

## Discussion

Traditional Chinese Medicine (TCM) has been used in a range of medical practices and health interventions in China and throughout the world, and body constitution is a foundational principle of TCM (6). The concept of body constitution centers on an individual’s predisposition to certain diseases, encompassing both physical conditions and psychological disorders (1, 2).

The aim of our study is to explore the relationship between the nine TCM-based body constitutions and the mental health of model workers in Beijing. Our findings seek to offer guidelines that could enhance the quality of life, health support, and overall well-being of this exceptional and representative group. Our research results indicate that we have successfully achieved this objective. According to our knowledge, we are the first to perform such an investigation into body constitutions of model workers and their mental health. Previous studies have primarily concentrated on the TCM body constitutions of various professions or university students and their associations with mental health (2).

Identification of body constitutions and the potential mental illnesses they may be associated can be beneficial in treating such illnesses. For example, recognizing body constitutions that are susceptible to depression and anxiety can aid in the prevention and treatment of these disorders (15–18). We have identified such associations in this study. According to **Table 2**, the Yang-Deficiency and Qi-Deficiency constitutions show a significant positive association with the SCL-90, particularly in the domains of depression, anxiety, and paranoid ideation. This finding agrees with previous research that also demonstrated Yang-Deficiency and Qi-Deficiency constitutions are indeed associated with depression (15, 16). In contrast, the Balanced constitution demonstrated a notable negative correlation with all SCL-90 domains, which makes sense because these individuals are not deficient in any category, thus according to TCM theory, they should not be prone to depression or anxiety (15). This result is also confirmed in the PLS modeling (**Figure 1**). As for the other constitutional types, they all displayed relatively weaker or non-significant correlations with the SCL-90 (**Table 3**).

Furthermore, Traditional Chinese medicine believes that different types of constitution reflect an individual’s physiological and psychological characteristics (19). Within TCM body constitution theory, Yang-Deficiency and Qi-Deficiency constitutions are typically linked to depression, given their close association with mental health issues (6, 8). In contrast, Balanced constitution is considered a relatively healthy type of constitution, as these individuals do not have obvious physical defects and are less prone to symptoms of depression or anxiety (6). Additionally, individuals with a Balanced constitution typically have a strong physique, are adaptable to environmental changes, and are less prone to illness (5). Other studies have found that a higher proportion of patients suffering from depression exhibit one of the eight unbalanced body constitution types (20–24).

Moreover, according to the PLS model (**Figure 1**) from our research demonstrates a relatively strong correlation between the Balanced constitution and elevated levels of psychological well-being, with a path coefficient of -0.503. This indicated that Balanced constitution has a higher level mental health status, and it certainly stands to reason that only Balanced constitution would lead to a state of complete psychological well-being. Additionally, this is in accordance with previous traditional Chinese medicine research literature (25). In contrast, the other eight constitutional types exhibit path coefficients exceeding 0.3, indicating a tendency toward lower levels of psychological well-being and this is also in agreement with previous research (14, 15).

This study has several limitations which must be acknowledged. First, this study’s population is purely based on model workers in Beijing. Even though model workers consist of a special group of workers within China, as they are the most outstanding workers within their chosen professions, they nevertheless represent a very limited group of high-achieving individuals. This limits the best approximation of a population. Second, this study selected research subjects from a single hospital in Beijing. This is a geographical limitation. Multi-center studies from a variety of geographical regions should be conducted in the future.

## Conclusion

In conclusion, the results of this investigation underscore that the Balanced constitution continues to be closely associated with higher levels of psychological well-being, while the remaining eight constitutional types are consistently linked to lower levels of psychological well-being.

## Data Availability

The data analyzed in this study is subject to the following licenses/restrictions: patient data. Requests to access these datasets should be directed to HC, chl0227@mail.ccmu.edu.cn.
